# Therapy of polycythemia vera: is it time to change?

**DOI:** 10.18632/oncotarget.22282

**Published:** 2017-11-03

**Authors:** Francesco Passamonti, Margherita Maffioli, Barbara Mora

**Affiliations:** Francesco Passamonti: Department of Medicine and Surgery, Hematology, University of Insubria, Varese, Italy and Ematologia, ASST Sette Laghi-Ospedale di Circolo, Varese, Italy

**Keywords:** polycythemia, JAK2, ruxolitinib, interferon

Polycythemia vera (PV) is a myeloproliferative neoplasm (MPN) driven by *JAK2* mutations in most cases.[1] The disease has an increased risk of thromboembolic complications and a predisposition to evolve into post-PV myelofibrosis (PPV-MF). The updated World Health Organization (WHO) diagnostic criteria for PV have been published in 2016.[1] The most impactful modifications have been the lowering of the hemoglobin threshold to 16.5 g/dL in males and 16.0 g/dL in females, and the introduction of bone marrow morphology as a major criterion. Patients older than 60 years of age or with a previous thrombotic event are considered at high risk for thrombosis.[2] Leukocytosis and high *JAK2*V617F allele burden are additional risk factors for the occurrence of thrombosis and for progression to PPV-MF, respectively.[3] Once PV progresses to PPV-MF, it presents with a specific clinical picture.[4] At that time, survival must be assessed on the basis of the recently developed MYSEC-PM (MYelofibrosis SECondary-Prognostic Model).[5]

Therapy is based on phlebotomy to maintain the hematocrit below 45% and aspirin.[2] When cytoreduction is necessary (high risk for thrombosis and hyper-myeloproliferation), hydroxyurea (HU) is the first line treatment in most countries.

Is it time to move from HU as first line treatment? At the last meeting of the American Society of Hematology two phase 3 prospective trials on first line treatment of PV have been presented, both having complete hematological remission at 12 months as primary endpoint and including treatment-naïve or newly diagnosed patients (http://www.bloodjournal.org/content/128/22). The PROUD PV study is a randomized (ropeginterferon-alfa vs. HU), non-inferiority study conducted in 257 patients. Overall, 45% of patients reached the endpoint, without significant differences between the two treatments. The MPD-RC 112 trial compared peg-interferon with HU in 168 patients. Complete, partial and overall response rate were observed in 33%, 36% and 69% for HU-treated patients and in 28%, 53% and 81% for peg-IFN-treated patients, without statistically significant differences between the two arms. Grade 3 toxicity occurred in 14% and 44% of patients treated with of HU and peg-IFN, respectively. Thus, given the currently available information, it seems that there is no indication to move from HU as first line therapy for PV. We suggest considering interferon treatment in high-risk females of childbearing potential and in high-risk young patients who refuse HU.

Having decided the first line treatment and the goals for the patient’s follow-up, the identification and management of patients who are intolerant/resistant to HU is critical. Criteria for HU intolerance and resistance have been proposed, but these were generated for clinical trials and not for clinical practice. So, are these criteria useful in the real life to capture patients with unfavorable outcome? A Spanish collaborative study on 890 PV patients found that these criteria were present in 15% of them,[6] in detail: need for phlebotomies (3.3%), uncontrolled myeloproliferation (1.6%), failure to reduce massive splenomegaly (0.8%), cytopenia at the lowest HU-dose to achieve response (1.7%), extra-haematological toxicity (9%). Concerning the predictive value of these criteria, cytopenia affected survival, progression to PPV-MF and blast phase and splenomegaly affected PPV-MF.

Two prospective randomized studies, named Response (including 222 patients)[7] and Response -2 (including 173 patients)[8] evaluated PV patients with HU intolerance/resistance in need of phlebotomy with (Response) or without (Response-2) splenomegaly. Patients were randomized to receive ruxolitinib or best available therapy (BAT). Primary composite endpoints were hematocrit control in the absence of phlebotomy (both studies) and 35% reduction in spleen volume at week 32 (Response). In the Response trial, 21% of ruxolitinib-treated patients and 1% of BAT-treated patients achieved the primary endpoint. Hematocrit control was achieved in 60-62% of patients in the two studies; 38% of ruxolitinib-treated patients obtained the spleen endpoint. The most frequent hematological adverse events of any grade were anemia (14% in the ruxolitinib group vs. 3% in the BAT group) and thrombocytopenia (3% vs. 8%, respectively).

These studies indicate that doctors can consider ruxolitinib as second-line therapy after HU failure/intolerance with the aim of controlling hematocrit and/or splenomegaly. However, some words of caution are needed. First, hematocrit is a surrogate endpoint of thrombosis. It is of interest that in the 80-weeks follow-up analysis of the Response study, the rate of all thrombotic events (any grade) was 1.8 x 100 patient-years of exposure to ruxolitinib and 8.2 x 100 patient-years of exposure to standard care.[7] Second, available follow-up is insufficient to assess the effect of ruxolitinib on PPV-MF evolution. Third, which are the patients with HU resistance/intolerance who are really in need of second line treatment? For instance, in a patient receiving a few phlebotomies along with HU to maintain the hematocrit below 45% ruxolitinib might be superfluous. On the other hand, patients with spleen enlargement, symptomatology, uncontrolled myeloproliferation despite HU or who are intolerant to HU might be candidates to ruxoltinib in real life (within the EMA/FDA label).

In the setting of second line treatment in PV, other therapies are under investigations. Restoration of p53 activity inhibiting the p53-MDM2 interaction is an attractive approach and is under investigation in HU-resistant/intolerant PV patients (RG7388, a second generation MDM2 inhibitor - NCT02407080). TGR-1202 is an orally available PI3K delta inhibitor, targeting the delta isoform. A combination with ruxolitinib is under way in MPN incuding HU-resistant PV (NCT02493530).

In conclusion, the treatment scenario of PV is moving forward with HU and potentially interferons to be considered as first line and ruxolitinib being active and safe in patients failing HU.
